# Phosphoregulation of the oncogenic protein regulator of cytokinesis 1 (PRC1) by the atypical CDK16/CCNY complex

**DOI:** 10.1038/s12276-019-0242-2

**Published:** 2019-04-16

**Authors:** Sara Hernández-Ortega, Abril Sánchez-Botet, Eva Quandt, Núria Masip, Laura Gasa, Gaetano Verde, Javier Jiménez, Rebecca S. Levin, Florentine U. Rutaganira, Alma L. Burlingame, Don Wolfgeher, Mariana P. C. Ribeiro, Stephen J. Kron, Kevan M. Shokat, Josep Clotet

**Affiliations:** 1 Faculty of Medicine and Health Sciences, International University of Catalonia, 08195 Sant Cugat del Vallès Barcelona, Spain; 2 Department of Cellular and Molecular Pharmacology and Howard Hughes Medical Institute, University of California, San Francisco, CA 94158 USA; 3 Department of Pharmaceutical Chemistry, University of California, San Francisco, CA 94158 USA; 4 Department of Molecular Genetics and Cell Biology, The University of Chicago, Chicago, IL 60637 USA

**Keywords:** Protein-protein interaction networks, Cell division, Cytoskeleton, Protein-protein interaction networks, Cell division

## Abstract

CDK16 (also known as PCTAIRE1 or PCTK1) is an atypical member of the cyclin-dependent kinase (CDK) family that forms an active complex with cyclin Y (CCNY). Although both proteins have been recently implicated in cancer pathogenesis, it is still unclear how the CDK16/CCNY complex exerts its biological activity. To understand the CDK16/CCNY network, we used complementary proteomic approaches to identify potential substrates of this complex. We identified several candidates implicating the CDK16/CCNY complex in cytoskeletal dynamics, and we focused on the microtubule-associated protein regulator of cytokinesis (PRC1), an essential protein for cell division that organizes antiparallel microtubules and whose deregulation may drive genomic instability in cancer. Using analog-sensitive (AS) CDK16 generated by CRISPR-Cas9 mutagenesis in 293T cells, we found that specific inhibition of CDK16 induces PRC1 dephosphorylation at Thr481 and delocalization to the nucleus during interphase. The observation that CDK16 inhibition and PRC1 downregulation exhibit epistatic effects on cell viability confirms that these proteins can act through a single pathway. In conclusion, we identified PRC1 as the first substrate of the CDK16/CCNY complex and demonstrated that the proliferative function of CDK16 is mediated by PRC1 phosphorylation. As CDK16 is emerging as a critical node in cancer, our study reveals novel potential therapeutic targets.

## Cancer: Proteins that work together could reveal drug targets

Studying the activity of proteins that work together to control cell division is revealing several that might be suitable targets for new drugs to fight cancer. Researchers led by Josep Clotet and Mariana Ribeiro at the International University of Catalonia, Barcelona, Spain, investigated the activities of the complex formed between two proteins, CDK16 and CCNY. CDK16 is an enzyme that modifies other molecules by adding phosphate groups (PO_4_) to them. CCNY is a protein that controls the activity of CDK16 and other proteins. Previous research has suggested a role for the complex in the development of cancer, but the mechanism has been unclear. The researchers found that the CDK16/CCNY complex activates proteins that control the network of microtubules in cells known as the cytoskeleton. One of these proteins, PRC1, is essential for cell division.

## Introduction

Cyclin-dependent kinase 16 (CDK16, also known as PCTAIRE1 or PCTK1) is a member of the PCTAIRE family, which is a group of kinases related to the CDK family that includes PCTAIRE-1, −2 and −3^1^. CDK16 is broadly expressed in human tissues, with the highest levels found in brain and testis^2^. High levels of CDK16 expression can also be found in a wide range of transformed and immortalized cell lines of epithelial origin^3–5^. CDK16 has been implicated in cell proliferation, neurite outgrowth, myogenesis, protein secretion, vesicular exocytosis, spermatogenesis and spindle orientation^6–13^. Moreover, CDK16 plays an oncogenic role that has been linked to the regulation of tumor suppressor p27 protein degradation^14^, activation of the mammalian target of rapamycin pathway^15^ and regulation of the WNT/β-catenin pathway^16^. Interestingly, it was recently shown that the small molecule kinase inhibitor dabrafenib (Tafinlar) not only targets oncogenic mutant BRAF V600E but also potently inhibits CDK16^17^ and that this mechanism may contribute to the efficacy of this drug against *BRAF* wild-type (WT) tumors^18^.

To control progression through the cell cycle, CDKs must interact with their regulatory partners, the cyclins^19^. Recent studies identified cyclin Y (CCNY) as a key cyclin binding partner of CDK16 and demonstrated its ability to promote a 100-fold increase in the catalytic activity of CDK16^7,20^. On the other hand, CDK16 phosphorylates CCNY, which may serve as a mechanism activating the complex^20^. Overexpression of CCNY enhances the proliferation of glioma^21^ and ovarian cancer cells^22^, suggesting that CCNY is also implicated in cancer development and progression.

The protein regulator of cytokinesis 1 (PRC1) binds and organizes antiparallel microtubules, playing a key role in the execution of the ordered events that occur during mitosis and cytokinesis^23–26^. The exact role of PRC1 phosphorylation at Thr470 and Thr481 by CDK1/CCNB in early mitosis is still under debate, but it seems to be essential for the scheduled interaction with the motor protein Kif4^27,28^, timely assembly of the central spindle^29^, and timely binding to Plk1^30^. Interestingly, the abovementioned threonine residues are located in a nuclear localization signal (NLS) region^31^, suggesting that they might play a role in the regulation of PRC1 localization during interphase; however, it is still unknown whether other CDKs are able to phosphorylate PRC1 during interphase. Importantly, PRC1 overexpression appears to promote human carcinogenesis, as demonstrated in breast^32^, bladder^33^, liver^34^, pancreatic^35^ and gastric cancers^36^.

Whereas both CDK16 and CCNY have been implicated in cell proliferation and cancer, the physiological substrates of the CDK16/CCNY complex have yet to be identified. Here, using unbiased proteomic approaches, we revealed PRC1 as the first *bona fide* substrate of the CDK16/CCNY complex. Moreover, using a 293T analog-sensitive (AS) CDK16 clonal cell line generated by CRISPR-Cas9 that allows specific CDK16 inhibition, we verified that CDK16 inhibition leads to PRC1 delocalization to the nucleus. Moreover, our results suggest that the proliferative action promoted by CDK16 is mediated by PRC1, unveiling a new mechanism of PRC1 regulation that may contribute to tumor initiation and progression.

## Materials and methods

### Plasmids and recombinant proteins

cDNA of human CDK16, CCNY and PRC1 was amplified with an N-terminal GST fusion tag from 293T cells and cloned into the pGEX6P1 vector (GE Healthcare Life Sciences, Little Chalfont, UK). Site-directed mutagenesis of wild-type GST-CDK16 and GST-PRC1 sequences was performed to obtain the analog-sensitive CDK16 (F240G, AS-GST-CDK16) and PRC1-T481A constructs. For expression, plasmids were transformed into BL21 DE3 cells (Bio-Rad, Hercules, CA, USA). An overnight culture was used to inoculate (1:500) 1 L of LB medium containing 50 μg/ml ampicillin, and cells were incubated at 37 °C to an OD600 between 1.0 and 1.3; at this point, cells were placed at 16 °C and treated overnight with 0.2 mM isopropyl β-D-1-thiogalactopyranoside. Cells were harvested the following day and resuspended in lysis buffer (50 mM Tris, pH 7.5; 1 M NaCl; 1 mM MgCl_2_; 10% glycerol; 5% Triton X-100; and 1 mM DTT) and lysed with a microfluidizer. After the cell debris was pelleted, the lysate was loaded onto a column containing glutathione Sepharose (Amersham, GE Healthcare Life Sciences) for 4 h at 4 °C. The column was equilibrated with wash buffer (50 mM Tris, pH 8; 150 mM NaCl; 1 mM MgCl_2_; 10% glycerol; and 1 mM DTT), washed and eluted with 5 ml of wash buffer supplemented with 20 mM glutathione (Sigma-Aldrich, St. Louis, MO, USA). The eluate was concentrated with Amicon centrifugal filters (Millipore), and the protein was then purified by size exclusion chromatography using Superdex200 gel filtration columns (GE Healthcare Life Sciences). Finally, the desired fractions were collected and concentrated to a final concentration of 10 mg/ml.

The CDK1/CCNB complex was obtained from Sigma-Aldrich, and the MBP protein was purchased from Merck Millipore (Darmstadt, Germany).

### In vitro kinase assays

Purified WT-GST-CDK16 or AS-GST-CDK16 (0.5 µM) and GST-CCNY (5 μM) were added to purified GST-PRC1 in a solution containing 50 mM HEPES, pH 7.5; 10 mM MgCl_2_; and 1 mM DTT. ATPγS (250 μM) was then added for 1 h at room temperature. Reactions were quenched by adding EDTA (50 mM) and further incubated with 0.4 mg/ml paranitrobenzomesylate for 30 min at room temperature. The paranitrobenzomesylate-labeled lysate was then boiled with SDS sample buffer, and the samples were probed for thiophosphorylation by western blotting.

Alternatively, ATP (10 μM) and [γ-^32^P]-ATP (0.5 μCi/μl) were added to each sample to start the reaction. Upon completion of the reaction, 3 μl of the reaction mixture was spotted onto P81 paper. The paper was dried for 5 min under a heat lamp, washed 1 time for 30 s and 6 times for 5 min each in 1% phosphoric acid and dried completely for 15 min under a heat lamp. The paper was exposed overnight to a phosphor screen imaged on a Typhoon 9500, followed by quantification of the band intensity using densitometry. IC_50_ values were estimated by quantifying two independent experiments performed in duplicate, and the dose response was determined by fitting the data to a sigmoid function in Prism 7.0 (GraphPad Software) and reported as mean values.

For gel loading, the reactions were quenched by adding gel loading buffer and heating to 90 °C for 10 min before SDS–PAGE separation. Gels were washed, dried and detected by autoradiography.

### Cell lines and reagents

Cells were cultured in Dulbecco’s modified Eagle’s medium (Sigma-Aldrich) supplemented with 10% fetal bovine serum (Sigma-Aldrich), 1% GlutaMAX (Biowest, Nuaillé, France) and 1% penicillin/streptomycin (Sigma-Aldrich). All cells were grown in a humidified atmosphere at 37 °C and 5% CO_2_. Mycoplasma contamination was monitored periodically.

Staurosporine and the staurosporine analog Star 12 ((7S)-12-(4-aminobutyl)-7-(2-methylpropyl)-6,7,12,13-tetrahydro-5H-indolo[2,3-a]pyrrolo[3,4-c]carbazol-5-one) were synthesized as described^37^. PP1 (4-amino-5-(methylphenyl)-7-(t-butyl)pyrazolo-(3,4-d)pyrimidine), 3MBPP1 (1-(tert-butyl)-3-(3-methylbenzyl)-1H-pyrazolo[3,4-d]pyrimidin-4-amine) and the other PP1 analogs were synthesized as described^38^.

### CCNY interactome

MCF7 cells were scraped with lysis buffer containing 25 mM Tris, pH 7.4; 150 mM NaCl; 1 mM EDTA; 0.2–1% octylphenoxypolyethoxyethanol; and protease and phosphatase inhibitors (Thermo Scientific, Waltham, MA, USA). Lysates were incubated for 15 min on ice and sonicated, and cell debris was precipitated by centrifugation at 14,000 rpm for 15 min at 4 °C. Protein concentrations were determined by the Bradford assay (Bio-Rad). Five micrograms of an affinity-purified rabbit polyclonal anti-CCNY antibody (18042–1-AP, Proteintech, Rosemont, IL, USA) or IgG as the negative control was crosslinked with disuccinimidyl suberate crosslinker (Thermo Scientific) to 20 μl of Protein A magnetic beads (Thermo Scientific). The beads were then incubated with 3–5 mg of MCF7 cell lysate for 14–15 h at 4 °C. The beads were washed twice with lysis buffer and once with deionized water. Proteins were eluted with gel loading buffer for 10 min at 30 °C. Proteins were separated by electrophoresis and gels stained with Imperial Protein Stain (Thermo Scientific).

Samples were subjected to in-gel trypsinization, and peptides were analyzed by LC–MS/MS. All LC–MS/MS *.raw data files were analyzed with MaxQuant version 1.5.2.8, searching against the SPROT 150220_SPROT_Human_Iso database downloaded on February 20, 2015, using the following criteria: LFQ quantification with a minimum of 1 high-confidence peptide. Trypsin was selected as the protease, with the maximum missed cleavage parameter set to 2. Carbamidomethyl (C) was selected as a fixed modification. Variable modifications were set to deamidation (NQ), oxidization (M), formylation (n-term), and phosphorylation (STY). An Orbitrap mass spectrometer was selected, using an MS error of 20 ppm and an MS/MS error of 0.5Da. An FDR cutoff of 1% was selected for peptide, protein, and site identifications. FQ intensities were reported based on the MS level peak areas determined by MaxQuant and reported in the proteinGroups.txt file. Three complete biological replicates were performed. Statistical and differential expression analyses were performed in R with the DEP package. Briefly, the LFQ intensities were used as the protein abundances, and contaminants and reverse proteins were removed. The data were imputed with the “QIRLC” setting and normalized. The data were filtered at a p-value cutoff of 0.05 and a fold change cutoff of log_2_(3.23) and observed in all three biological replicates. The mass spectrometry proteomic data were deposited to the ProteomeXchange Consortium (http://proteomecentral.proteomexchange.org) via the PRIDE partner repository^39^. All plots were generated with the DEP package^40^.

### Capture and identification of thiophosphorylation substrates

HeLa cells were lysed with a sonicator in pH 7.5 HEPES buffer. Lysates were labeled for 1 h at room temperature in a reaction mixture containing 2 mg of lysate; 500 nM WT-GST-CDK16 or AS-GST-CDK16; 5 μM GST-CCNY; 250 μM N^6^-phenetyl ATPγS (Axxora, Farmingdale, NY, USA); 250 μM ATP; 50 mM HEPES, pH 7.5, 10 mM MgCl_2_; 1 mM DTT; and 3 mM GTP to block nonspecific labeling. Control experiments without kinase were performed in parallel. The reaction was quenched with 50 mM EDTA.

The thiophosphate-labeled sample was enriched and analyzed by tandem mass spectrometry (LC–MS/MS) as previously described^41^. Briefly, samples were denatured with urea and cleaved with MS-grade trypsin (Promega, Madison, WI, USA) overnight at 37 °C. Peptides were then purified on a SepPak column and enriched by binding to SulfoLink iodoacetyl resin (Pierce) overnight at room temperature in the dark. Finally, samples were washed and eluted with potassium peroxymonosulfate (Oxone) and purified by Zip-tip (Millipore). Desalted peptides were resuspended in 10 μl of 0.1% formic acid. Peptides were then loaded into a nanoACQUITY (Waters) UPLC instrument for reversed-phase chromatography with a C18 column (BEH130, 1.7μm bead size, 100 μm × 100 mm) in front of an LTQ Orbitrap Velos. The LC instrument was operated at a 600 nl/min flow rate, and peptides were separated over an 80 min gradient from 2 to 50% Buffer B (Buffer A: water and 0.1% formic acid, Buffer B: acetonitrile and 0.1% formic acid). Survey scans were recorded over a 350–1800 m/z range, and MS/MS fragmentation was performed using ETD on the top 8 peaks. Peak lists were generated with the UCSF program PAVA and searched against the SwissProt *Homo sapiens* database (downloaded June 27, 2013, 20 264 entries) using Protein Prospector (version 5.10.10). The data were screened with a 20 ppm tolerance for parent and fragment ions, allowing for standard variable modifications and S/T/Y phosphorylation. The background peptides and phosphopeptides were filtered using an in-house R script as previously described^42^.

### Analog-sensitive (AS)-CDK16 cell line

The pSpCas9(BB)-2A-Puro (pX459) V2.0 vector was obtained from Addgene (Cambridge, MA, USA; plasmid #62988). The CDK16-specific guide RNA (gRNA) and the single-stranded (ssDNA) repair template were selected using the design tool DESKGEN and purchased from IDT. The gRNA was cloned into pX459 as described elsewhere^43^. 293T cells were cotransfected with 1 µg of gRNA plasmid DNA and the ssDNA donor (final concentration, 10 µM) using FuGENE HD (Promega). After 8 h, SCR-7 (1 µM, Xcess Biosciences Inc., San Diego, CA, USA) was added to the medium to suppress nonhomologous end-joining repair^44^. Transfected cells were then cultured in medium containing 1 μg/ml puromycin for 3 days for selection. Surviving cells were trypsinized, and clones were isolated by dilution. Each colony was genotyped, and insertion of the desired mutation was confirmed by sequencing.

### Colony formation assay

For this assay, 293T AS-CDK16 and control cells were plated in 6-well plates at a density of 1000 cells per well and treated with 0, 5 or 10 µM 3MBPP1. MCF7 (1 700 cells), HeLa (1200 cells) and HT-29 (1500 cells) cells were seeded in 6-well plates 3 days after transfection with siRNA targeting CDK16 and/or PRC1 or with scrambled control siRNA. Colonies became visible after two weeks and were fixed with 100% cold methanol, washed with PBS and stained with 0.1% crystal violet for 30 min at room temperature in the dark. The number of colonies was determined, and survival was calculated as the percentage of the control.

### Drug treatment

HT-29 cells were seeded in 6-well plates at a density of 250,000 cells per well. The following day, cells were treated with dabrafenib (2 or 10 nM) for 24 h and harvested for western blot analysis.

### Cell extracts and immunoblot analysis

Thirty micrograms of total protein was separated by 15% SDS–PAGE. Proteins were then transferred to PVDF membranes (Immobilon-P, Millipore) and blocked with TBS-T (20 mM Tris, 137 mM NaCl, 0.1% Tween at pH 7.6) and 5% nonfat milk for 30 min at room temperature. After blocking, membranes were incubated with a specific primary antibody overnight at 4 °C. The following primary antibodies were used: 1:10000 rabbit monoclonal anti-thiophosphate ester (Epitomics, Inc., Burlingame, CA, USA), 1:1000 goat polyclonal anti-GST (GE Healthcare), 1:100 rabbit polyclonal anti-p27 (sc-528, Santa Cruz Biotechonology, Dallas, TX, USA) 1:1 000 rabbit polyclonal anti-PRC1 (sc-8356, Santa Cruz Biotechnology), 1:1000 anti-lamin B (sc-6216, Santa Cruz Biotechnology), 1:1000 anti-Na+/K+-ATPase (sc-58628, Santa Cruz Biotechnology) and 1:10,000 anti-GAPDH (CSB-PA00025A0Rb, Cusabio, San Diego, CA, USA). After several washes, membranes were incubated with horseradish peroxidase (HRP)-labeled secondary antibodies (Jackson Laboratories, West Grove, PA, USA) for 1 h at room temperature. After washing, bands were detected using Luminata Forte Western HRP Substrate (Millipore), and images were acquired using GeneSnap (Syngene) software. The density of the bands was analyzed using Image Studio Lite (Li-Cor) software, and Ponceau staining (Sigma-Aldrich) was used to normalize protein expression.

### Cell synchronization

To synchronize cells via double thymidine block, 24 h after seeding 293 T AS-CDK16 and control cells, the cells were treated with 2.5 mM thymidine for 16 h, released for 8 h, and blocked with 2.5 mM thymidine for another 16 h.

### Immunofluorescence

For immunofluorescence, 293T AS-CDK16 cells were plated in 24-well plates at a density of 25 000 cells per well. After synchronization by double thymidine block, 293T AS-CDK16 cells were released and treated with 0 or 5 µM 3MBPP1 for 6 h. HT-29 cells were plated in 24-well plates at a density of 30 000 cells per well, and 24 h later, cells were treated with dabrafenib (2 or 10 nM) for 24 h. After incubation, cells were fixed in 4% paraformaldehyde for 15 min at 4 °C. Next, cells were blocked with PBS containing 0.1% Triton X-100 and 10% horse serum for 1 h and were then incubated with 1:500 goat anti-pPRC1 (sc-11768, Santa Cruz Biotechnology) and/or rabbit anti-PRC1 (sc-8356, Santa Cruz Biotechnology) overnight at 4 °C. The coverslips were washed several times with PBS and incubated for 1 h at room temperature with anti-goat or anti-rabbit fluorescent secondary antibody at a 1:1000 dilution in the dark. After additional washes with PBS, nuclei were counterstained with Hoechst 33258 (Sigma-Aldrich), and the slides were coverslipped and imaged using a Nikon Eclipse Ti with a 60×objective. Image analysis was performed using NIS-Elements AR 4.13.04 software.

### Subcellular fractionation

Sequential lysis of cell membranes was performed as previously described^45^. Briefly, cells were trypsinized, harvested, resuspended in cold PBS and pelleted at 500 × *g* for 10 min at 4 °C. The supernatant was discarded, and the cells were resuspended in lysis buffer A (50 mM HEPES, pH 7.4; 150 mM NaCl; 1 M hexylene glycol; 25 µg/ml digitonin; and protease inhibitors) and incubated in a rotor for 10 min at 4 °C. The cells were centrifuged at 2000 × *g* for 10 min at 4 °C, and the supernatant, with the cytosolic fraction, was collected. Cold lysis buffer B was added (50 mM HEPES, pH 7.4; 150 mM NaCl; 1 M hexylene glycol; 1% octylphenoxypolyethoxyethanol; and protease inhibitors) and resuspended by vortexing. The extract was incubated on ice for 30 min and harvested at 7000 × *g* for 10 min at 4 °C. The supernatant collected contained the membrane-bound proteins. Cold lysis buffer C was added (50 mM HEPES, pH 7.4; 150 mM NaCl; 1 M hexylene glycol; 0.5% sodium deoxycholate; 0.1% sodium dodecyl sulfate; and protease inhibitors) supplemented with micrococcal nuclease (3U/ml) and incubated for 30 min in a rotor at 4 °C. The extract was pelleted at 7  800 × *g* for 10 min at 4 °C, and the resulting supernatant, containing the nuclear proteins, was collected.

### Small interfering RNA (siRNA)

For transfection, 293T AS-CDK16, MCF7, HT-29 and Huh7 cells were seeded in 24-well plates at a density of 25 000 cells per well, whereas HeLa cells were seeded at a density of 20000 cells per well. Cells were transfected the following day with PRC1 siRNA (5′-rCrGrCrUrGrUrUrUrArCrUrCrArUrArCrArGr.

UrArCrGrUGT-3′, IDT), CDK16 siRNA (#1472 and #1656, Ambion, Life Technologies, Carlsbad, CA, USA) or scrambled control (51-01-14-03, IDT) using Lipofectamine 2000 (12566014, Thermo Fisher Scientific).

### MTT assay

Five days after transfection, cells were incubated with MTT solution (Sigma) for 1 h at 37 °C. The resulting formazan crystals were dissolved in DMSO, and the absorbance was measured at 570 nm in a Synergy HT plate reader. Experiments were conducted in parallel with protein extraction and western blot analysis to confirm PRC1 downregulation.

### 5-Bromo-2′-deoxyuridine (BrdU) incorporation assay

Cell proliferation was monitored using a Cell Proliferation ELISA, BrdU, colorimetric kit (Roche). Three days after transfection, cells were incubated with BrdU labeling solution for 2 h. Cells were then fixed and DNA denatured, followed by incubation with a suitable anti-BrdU monoclonal antibody conjugated with peroxidase. For detection, the 3,3′,5,5′-tetramethylbenzidine (TMB) substrate was used, and the absorbance was measured at 370 nm using a Synergy HT plate reader.

### Statistical analysis

Data are presented as the means ± standard errors of the mean (SEMs), and statistical significance was determined using the Mann–Whitney test with the following categories for *P*-values: ***P < 0.001; **P < 0.01; and *P < 0.05. When the data were normally distributed, Student’s *T*-test was used. Statistical analyses were conducted using GraphPad Prism.

## Results

### CDK16/CCNY complex activity does not require interaction with other proteins

**Fig. 1:**
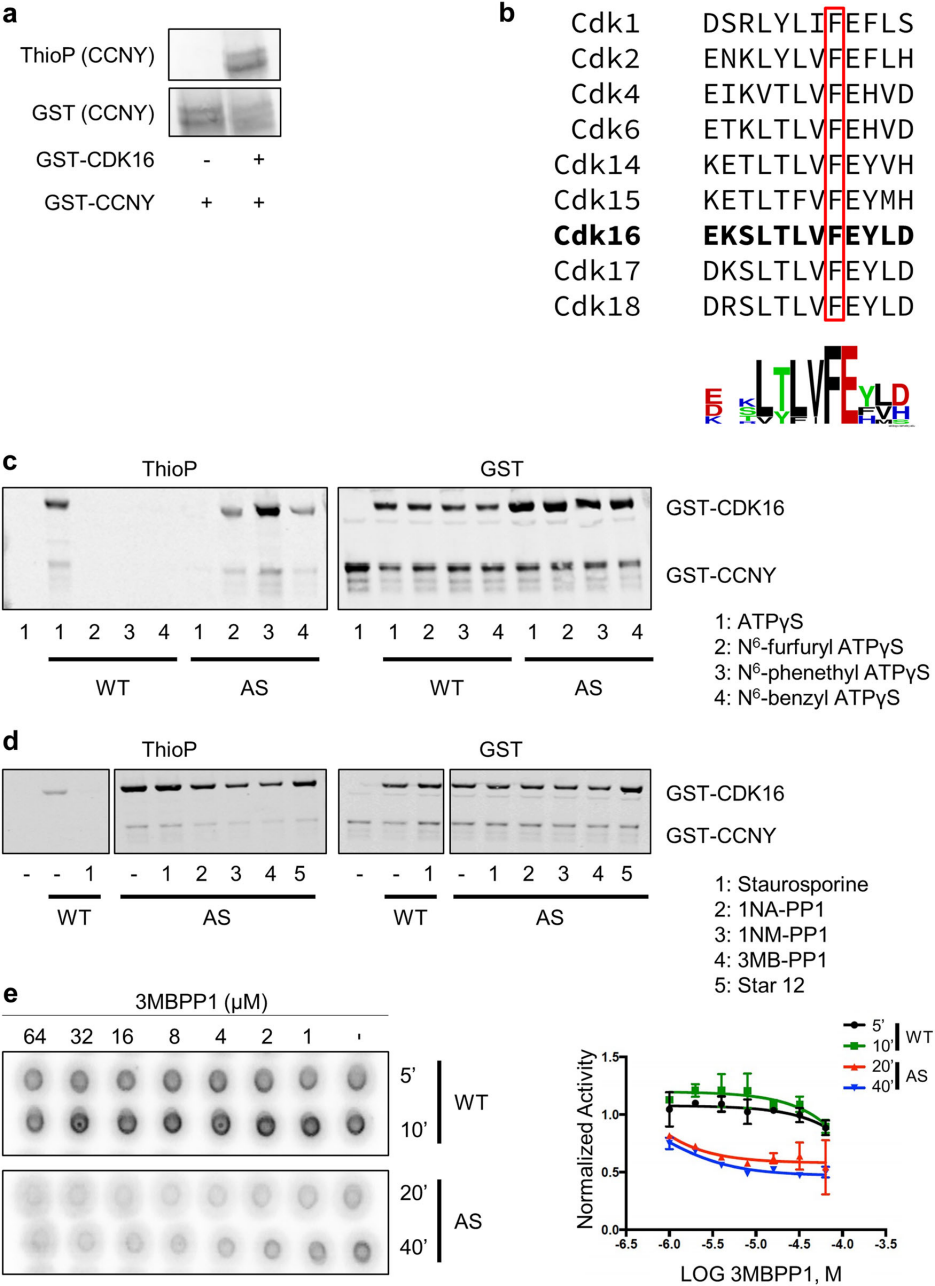
Generation of an Analog-Sensitive (AS) version of CDK16. **a** CDK16 phosphorylates CCNY in vitro. ATPγS was used as a phosphodonor, and thiophosphorylation (ThioP) was evaluated by western blotting. CDK16 and CCNY were purified from *E. coli*. **b** Identification of the gatekeeper residue in the ATP-binding pocket by sequence alignment with other CDKs. The motif for this conserved sequence was generated using WebLogo. **c** In vitro kinase assay using WT- or AS-GST-CDK16 with GST-CCNY as a substrate. The indicated N^6^-substituted ATPγS analog was used as a phosphodonor. **d** In vitro kinase assay using WT- or AS-GST-CDK16 with ATPγS or N^6^-Ph-ATPγS, respectively, in the presence of different inhibitors (1–5) to test the ability of these inhibitors to inhibit the AS mutant. **e** In vitro kinase assay conducted to establish the specific IC_50_ of 3MBPP1. WT- or AS-GST-CDK16 was incubated with [γ-^32^P]-ATP in the presence of different concentrations of 3MBPP1 for the indicated time intervals (in min)

To identify substrates of the CDK16/CCNY CDK–cyclin complex, we examined the activity of recombinant CDK16 and CCNY in an in vitro kinase assay. Initially, when the full-length, purified GST fusions were combined, no kinase activity was detected. However, eliminating protein aggregates by size exclusion chromatography yielded active complexes that displayed autophosphorylation of CDK16 (Supplementary Fig. S1), as well as phosphorylation of CCNY (Fig. 1a), consistent with the results of a previous study^20^. The complex was also able to weakly phosphorylate MBP (Supplementary Fig. S1). Thus, CDK16 can bind CCNY and display kinase activity independent of CDK -Activating Kinase (CAK) or the 14-3-3 protein.

### Generation of an Analog-Sensitive (AS) mutant of CDK16 that can use bulky ATP analogs and be specifically inhibited

Establishing the functional role of protein kinases in intricate signaling networks is often challenging. To characterize the biological actions of CDK16 and facilitate the identification of specific substrates, we generated a version of the WT kinase that can use orthogonal ATP analogs to identify direct substrates and can also be specifically inhibited with mutant-specific ATP-competitive inhibitors^46^. This chemical genetic approach allows the study of a single kinase (CDK16) while maintaining normal levels of protein expression and preventing the development of compensatory mechanisms by other CDKs.

Analog-sensitive forms of CDK1, CDK2, CDK7 and CDK9 have been developed previously^47–50^. The web-based application WebLogo^51^ was used to perform the alignment of the amino acid sequence of CDK16 and other PFTAIRE and PCTAIRE proteins with other members of the CDK family of kinases^52^. This analysis revealed that phenylalanine 240 is likely to be the gatekeeper residue on CDK16 (Fig. 1b). To generate an AS version of CDK16 (AS-CDK16), we mutated phenylalanine 240 to a glycine. We then tested the kinase activity of WT- and AS-CDK16 in vitro by incubating these kinases with recombinant GST-CCNY in the presence of ATPγS or a variety of N^6^-substituted forms of ATPγS. As expected, while WT-CDK16 used ATPγS to phosphorylate GST-CCNY, it could not use the N^6^-modified bulky forms of ATPγS (Fig. 1c). Accordingly, AS-CDK16 was less efficient using ATPγS than using the N^6^-substituted bulky forms of ATPγS, particularly N^6^-phenethyl ATPγS, to phosphorylate CCNY in vitro (Fig. 1c). This differential activity has already been described by others^53^. These findings support the hypothesis that phenylalanine 240 is the gatekeeper residue of CDK16 and that the mutation of this residue to a glycine allows CDK16 to use bulky analogs of ATP in vitro.

To identify an ATP-competitive inhibitor specific for AS-CDK16, we performed an in vitro kinase assay in which CCNY and either WT- or AS-CDK16 were incubated with the appropriate form of ATPγS analog in the presence of different PP1-based inhibitors. WT-CDK16 was inhibited by staurosporine (Fig. 1d), a well-known CDK family inhibitor, but it was not inhibited by any of the PP1-derived inhibitors tested. In contrast, AS-CDK16 was inhibited by neither staurosporine nor its derived inhibitor Star 12^37^, but it was inhibited to different levels by several PP1-derived inhibitors (Fig. 1d). Among these, 3MBPP1 was the most efficient at inhibiting both autophosphorylation and CCNY phosphorylation (Fig. 1d). Next, we monitored the specific inhibition of AS-CDK16 by 3MBPP1 in vitro to determine the IC_50_ value, as previously described^54^. Either WT or AS-CDK16 was incubated with [γ-P^32^]-ATP, with different optimal times for each kinase assay (Fig. 1e). While AS-CDK16 was fully inhibited by 5μM 3MBPP1 at an IC_50_ of 1.23μM, higher concentrations were needed to inhibit WT-CDK16 (IC_50_ = 4.4μM). In conclusion, AS-CDK16 can be selectively inhibited by 3MBPP1.

### Proteomic screening reveals candidate targets of CDK16/CCNY, including PRC1

**Fig. 2:**
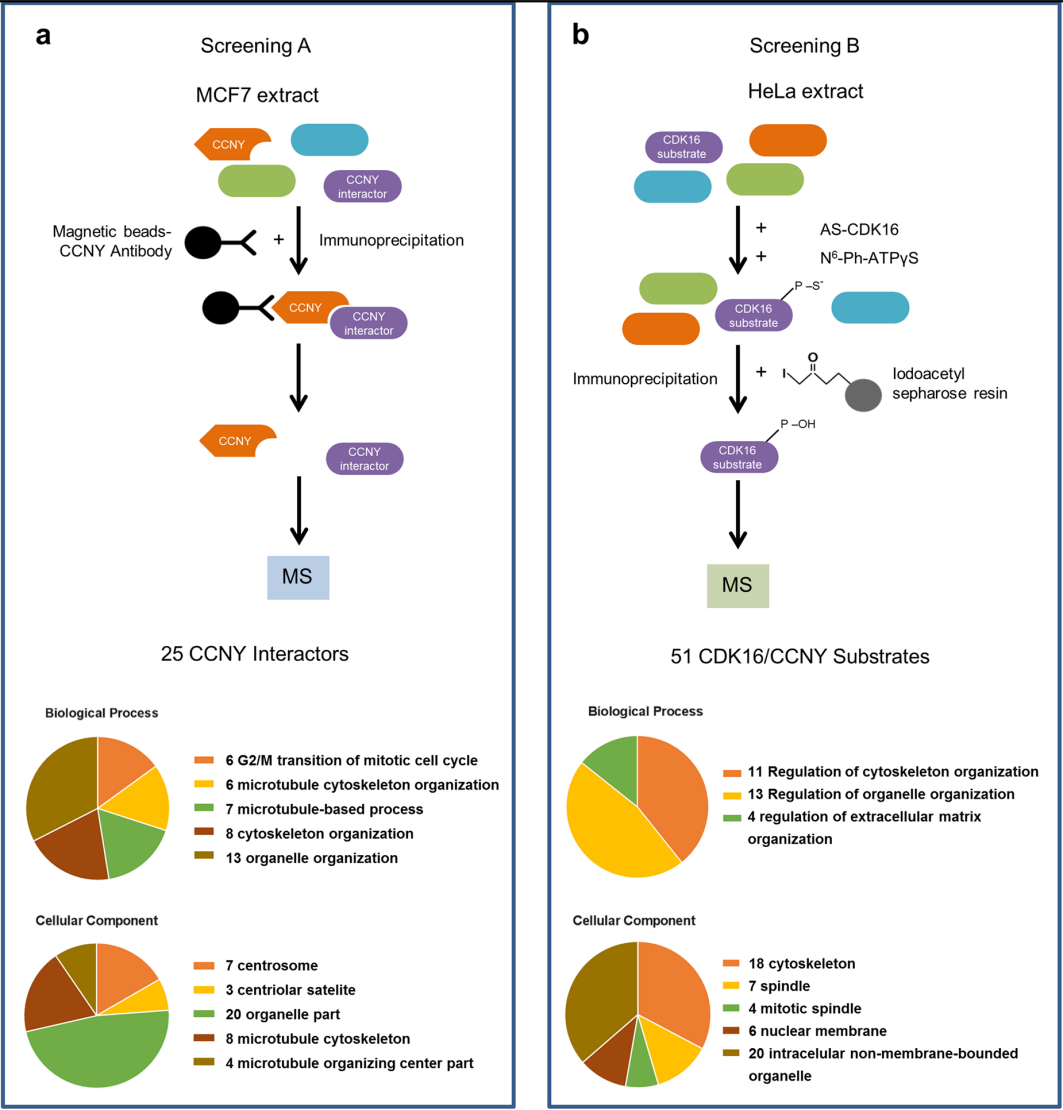
A double proteomic approach reveals a role for the CDK16/CCNY complex in cytoskeletal regulation. **a** Strategy used to identify CCNY interactors using MCF7 cell extracts. The graphical representation of GO terms obtained by STRING analysis for biological processes and cellular components is shown below. Data were filtered with a *p*-value cutoff of 0.05 and a fold change cutoff of log_2_(3.23) and seen in all three biological replicates. **b** Chemical genetic approach based on the labeling of AS-GST-CDK16 substrates in HeLa cell extracts. The corresponding graphical representation of GO terms obtained by STRING analysis is shown below. Peptides that were present in the AS-CDK16 proteomic analysis but in neither the WT-CDK16 nor the negative control analyses were selected. The significance threshold for the false discovery rate (FDR) was set at ≥0.01. MS, mass spectrometry; N^6^-Ph-ATPγS, bulky ATP analog

To discover physiological substrates of CDK16, we carried out two types of proteomic assays. First, we used affinity purification-mass spectrometry (AP-MS) to generate a CCNY interactome (Fig. 2a). Using anti-CCNY beads, we performed a pulldown of endogenous CCNY from a lysate of MCF7 cells—breast carcinoma cells where CCNY is expressed and plays an essential role^55^, isolated bound proteins with SDS–PAGE, and performed in-gel trypsinization. Using tandem mass spectrometry and LFQ quantification analysis via MaxQuant^56^, we identified several potential CCNY interactors. Among these candidate interacting proteins, 25 were selected as significant (Supplementary Table S1). Strikingly, gene ontology (GO) term enrichment analysis using the STRING database mapped many of the CCNY-interacting proteins to cytoskeleton-related pathways, with a false discovery rate of ≤0.01 (Fig. 2a).

**Fig. 3:**
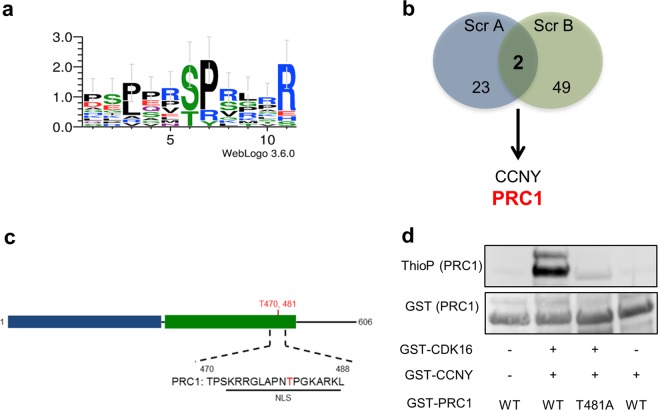
Identification of PRC1 as a direct substrate of the CDK16/CCNY complex. **a** The CDK16 phosphorylation consensus sequence was identified using WebLogo. **b** The Venn diagram of selected CCNY interactors (Screening A, Scr A) and CDK16/CCNY direct substrates (Screening B, Scr B) illustrates that PRC1 is the only substrate identified by both approaches. **c** Schematic representation of the human PRC1 locus showing the NLS domain where Thr481 is located. **d** In vitro kinase assay using WT-GST-CDK16, GST-CCNY and either WT or T481A-mutant of PRC1

In parallel, we applied a chemical genetic approach (Fig. 2b), which relies on adding recombinant GST-AS-CDK16/GST-CCNY complex and N^6^-phenethyl ATPγS to a cell lysate and then capturing thiophosphorylated peptides on iodoacetyl sepharose. The peptides, representing candidate phosphosites for the CDK16/CCNY kinase, were then identified by LC–MS/MS^41^. We conducted this analysis in HeLa cells to identify common substrates that are likely to be relevant in distinct genetic backgrounds. In addition to CDK16 and CCNY, 49 additional potential substrate proteins were identified that were present in neither WT-CDK16 cells nor in negative control cells (Supplementary Table S2). In agreement with the data from the CCNY interactome, several proteins in the list of candidate substrates were involved in cytoskeleton-related pathways (Fig. 2b). By selecting high-confidence hits (Supplementary Table S2), we generated a consensus sequence for phosphorylation by CDK16 (Fig. 3a), which was the prototypical sequence S/T-P, typical of all known CDKs (see discussion). This sequence suggests a preference for serine residues adjacent to prolines at the +1 position with an arginine at the +5 position. In addition, arginine was favored at any position from −1 to +4.

Only one protein other than CCNY was present in both lists (Fig. 3b)—protein regulator of cytokinesis 1, PRC1, a protein that crosslinks and organizes antiparallel microtubules in the midzone during cytokinesis. In the thiophosphorylation analysis, PRC1 was found to be modified on T481 in the peptide KRRGLAPNTPGKAR, within an NLS motif (Fig. 3c). Importantly, PRC1 nuclear localization is dynamic and has functional significance^34^. To validate PRC1 as a substrate, we expressed and purified GST-PRC1 and a GST-PRC1-T481A mutant and performed a kinase assay using the CDK16/CCNY complex and ATPγS. CDK16/CCNY strongly thiophosphorylated PRC1 but failed to modify the PRC1-T481A mutant (Fig. 3d).

### Generation of a 293T AS-CDK16 clonal cell line

**Fig. 4:**
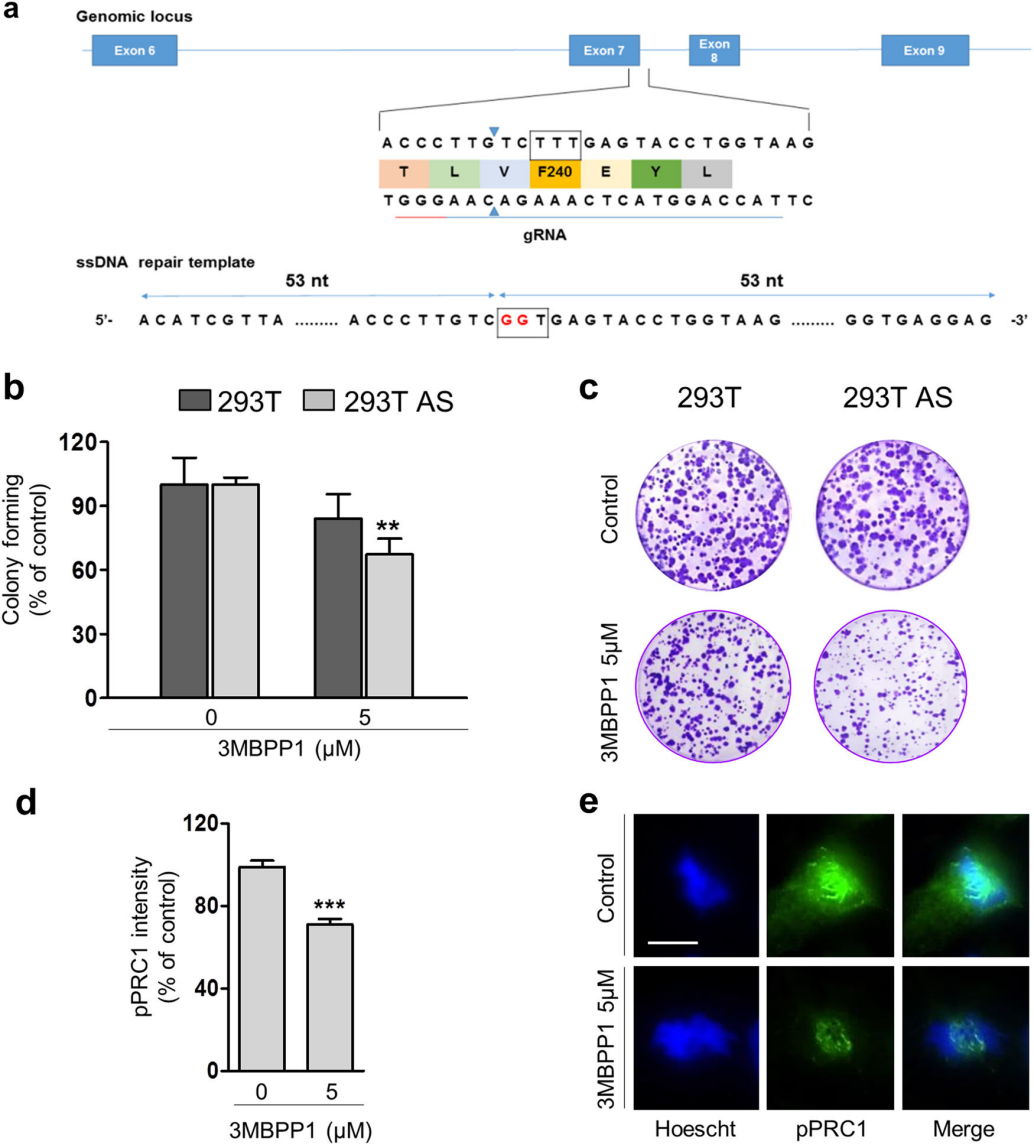
Generation of a clonal 293T analog-sensitive (AS)-CDK16 cell line by CRISPR-Cas9. **a** Schematic representation of the human CDK16 locus showing the target site used for CRISPR/Cas-mediated genome editing in 293T cells and the ssDNA used as repair template. (**b**, **c**) Colony formation assay of 293T and 293T AS-CDK16 cells grown in the absence or presence of 3MBPP1. The columns represent the means ± SEMs of 7 independent experiments performed in triplicate. **P < 0.01 vs nontreated cells, Mann–Whitney test. (**d,** **e**) 293T AS-CDK16 cells were synchronized and treated with 3MBPP1. After 6 h, cells were fixed and immunostained with a phospho-T481-PRC1 (pPRC1) antibody. Three independent experiments were performed, and 150–200 cells were analyzed per condition. The graph shows the results of one representative experiment, and the columns represent the means ± SEMs. ***P < 0.001 vs control, Student’s *t*-test

Our finding that PRC1 is a substrate of CDK16 is of particular interest, considering the roles of these two proteins in cancer cell proliferation^14,57^. To study CDK16-mediated regulation of PRC1 in vivo, we used CRISPR/Cas9 to introduce the F240G point mutation and generate a genomically stable 293T AS-CDK16 clonal cell line (Fig. 4a and Supplementary Fig. S2). Clonogenic assays showed decreased viability of 293T AS-CDK16 cells compared to that of WT cells after treatment with 3MBPP1 (Fig. 4b, c), indicating that using this model, we were able to reproduce phenotypes associated with CDK16 downregulation already described by others^14–16^. Consistent with the loss of CDK16^14^, we observed the accumulation of p27^Kip1^ upon treating 293T AS-CDK16 cells with 3MBPP1, whereas 3MBPP1 exhibited minimal effects on WT cells (Supplementary Fig. S3). We then examined PRC1 phosphorylation by CDK16 in vivo using 293T AS-CDK16 cells. 293T AS-CDK16 cells synchronized in G1 phase by double thymidine block were released in media and treated with 0 or 5 µM 3MBPP1. After 6 h, when most cells were in G2-M phase, the cells were fixed and immunostained with a rabbit monoclonal anti-phospho-T481 PRC1 antibody. This analysis showed decreased PRC1 T481 phosphorylation in 293T AS-CDK16 cells treated with 3MBPP1 (Fig. 4d, e). These findings provide validation of PRC1 as a CDK16 substrate and indicate that the 293T AS-CDK16 cell line and 3MBPP1 provide an approach to test functional significance.

### CDK16 regulates PRC1 nuclear localization and downregulation

**Fig. 5:**
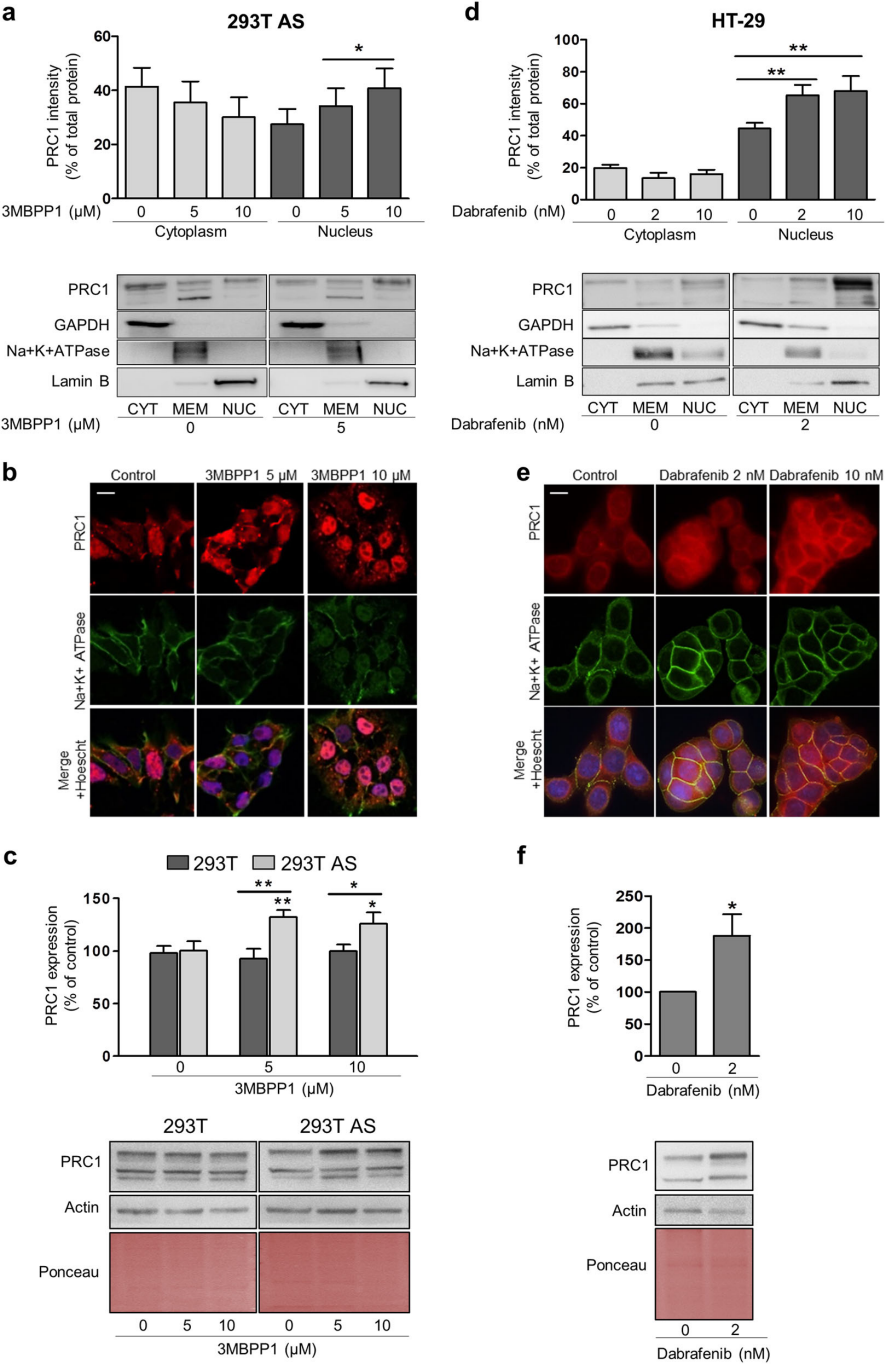
CDK16 inhibition promotes PRC1 delocalization to the nucleus and PRC1 accumulation. **a** Analysis of PRC1 distribution in 293T AS-CDK16 cells after 3MBPP1 treatment (0, 5 and 10 µM) and subcellular fractionation. The columns represent the means ± SEMs of 9 independent experiments. **b** Representative images of PRC1 immunofluorescence (red) and the Na+/K+ATPase (membrane marker) in 293T AS-CDK16 cells treated with 0 (control), 5 or 10 µM 3MBPP1. **c** PRC1 accumulation in 293T and 293T AS-CDK16 cells treated with 3MBPP1 (0, 5 and 10 µM) for 6 h was assessed by western blotting. The columns represent the means ± SEMs of 12 independent experiments. *P < 0.05, **P < 0.01 vs control; Mann–Whitney test. **d** Analysis of PRC1 distribution in HT-29 colon cancer cells after treatment with dabrafenib (0, 2 and 10 nM) for 24 h and subcellular fractionation. The columns represent the means ± SEMs of 8 independent experiments. **e** Representative images of PRC1 immunofluorescence (red) and the Na+/K+ ATPase in HT-29 cells treated with 0 (control), 2 or 10 nM dabrafenib for 24 h. **f** PRC1 accumulation in HT-29 cells treated with 2 nM dabrafenib for 24 h was assessed by western blotting. The columns represent the means ± SEMs of 8 independent experiments. *P < 0.05 vs nontreated (NT) cells, Mann–Whitney test

It was recently shown that the subcellular localization of PRC1 affects its functions in cell proliferation^34^. Considering that the CDK16 phosphosite on PRC1, T481, is embedded in a putative NLS, we examined whether PRC1 localization is modified by CDK16 phosphorylation. Extracts prepared from 293T AS-CDK16 cells grown in the presence of 0, 5 or 10 µM 3MBPP1 and subjected to subcellular fractionation revealed PRC1 accumulation in the nuclear fraction in a 3MBPP1 dose-dependent manner (Fig. 5a). This finding was confirmed by immunofluorescence analysis, which showed PRC1 relocalization to the nucleus in 293T AS-CDK16 cells treated with 10 µM 3MBPP1 (Fig. 5b). Considering that protein regulation is compartmentalized, we investigated the effect of CDK16 phosphorylation on PRC1 levels. Along with relocalization to the nucleus, 3MBPP1 treatment of 293T AS-CDK16 cells induced PRC1 accumulation (Fig. 5c). In contrast, in 293T WT-CDK16 cells, 3MBPP1 was unable to promote PRC1 accumulation (Fig. 5c). These observations were further confirmed by immunofluorescence studies, which showed that CDK16 inhibition decreased PRC1 phosphorylation, causing simultaneous PRC1 accumulation and nuclear localization (Supplementary Fig. S4).

The BRAF V600 mutant inhibitor dabrafenib^58,59^ is also a potent CDK16 inhibitor^18^. Accordingly, it promotes p27 accumulation in the *BRAF*-mutant colon cancer cell line HT-29 (Supplementary Fig. S3). Therefore, we investigated the effect of dabrafenib on PRC1 subcellular localization. In agreement with the results obtained in 293T AS-CDK16 cells, dabrafenib promoted a dose-dependent accumulation of PRC1 in the nuclear fraction, as demonstrated by western blot analysis (Fig. 5d) and immunofluorescence studies (Fig. 5e). Moreover, we observed that 2 nM dabrafenib promoted PRC1 accumulation in HT-29 cells (Fig. 5f). The similar induction of PRC1 accumulation and nuclear localization by a CDK16-specific inhibitor supports a role of CDK16 in regulating PRC1 function.

### CDK16-induced cell proliferation is dependent on the presence of PRC1

**Fig. 6:**
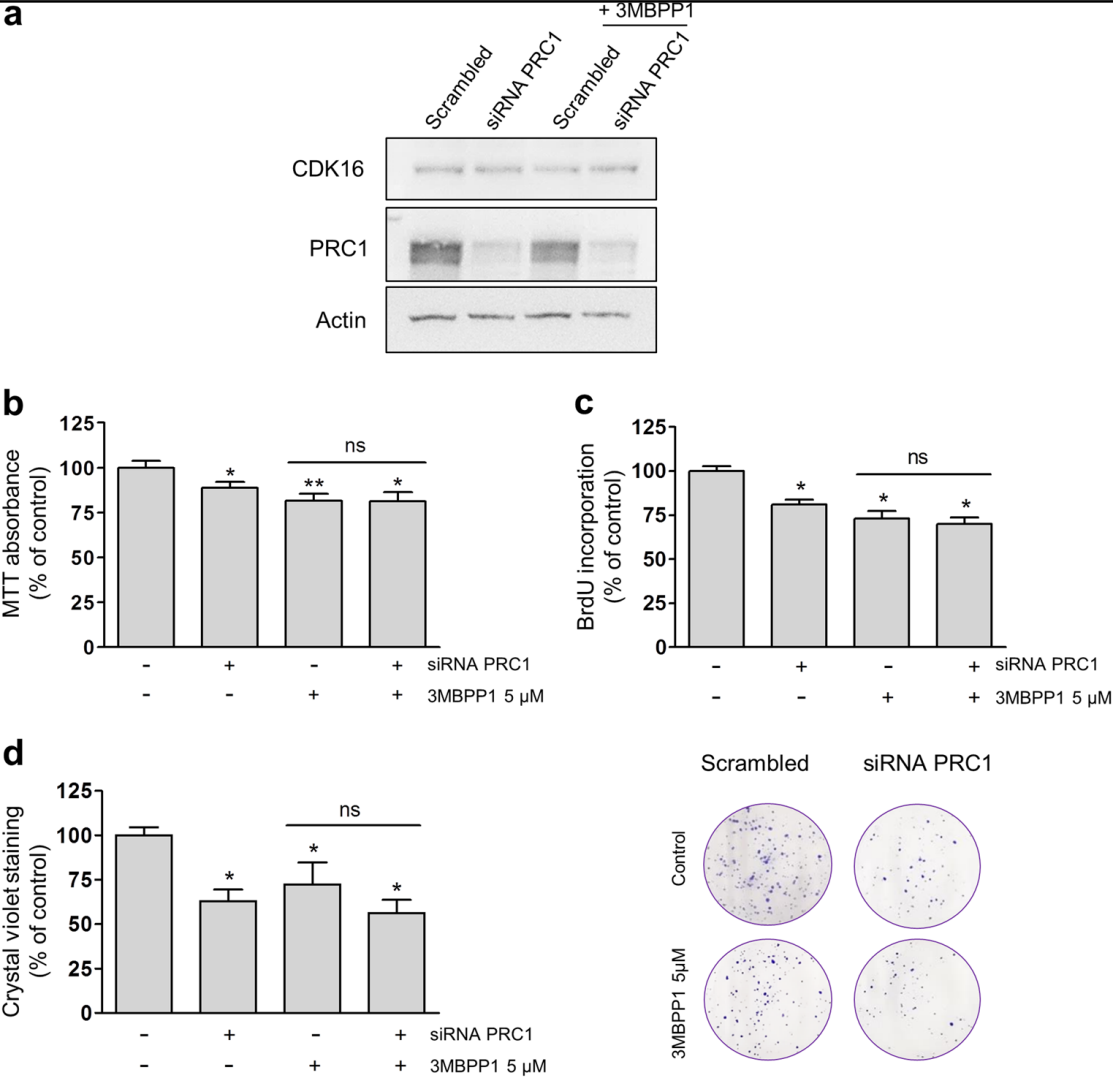
The proliferative action of CDK16 is mediated by PRC1 phosphorylation. **a**–**c** Here, 293T AS-CDK16 cells were seeded and transfected the following day with siRNA targeting PRC1 or with scrambled control and treated with 0 or 5 µM 3MBPP1. **a** Western blot analysis was performed to confirm PRC1 downregulation. **b** Cell viability was monitored by an MTT assay, and the columns represent the means ± SEMs of 6 independent experiments performed in triplicate. *P < 0.05, **P < 0.01 vs control; Mann–Whitney test. **c** Cell proliferation was assessed by a BrdU incorporation assay 72 h after transfection. The columns represent the means ± SEMs of 4 independent experiments performed in triplicate. *P < 0.05 vs control, Mann–Whitney test. **d** Here, 293T AS-CDK16 cells were seeded and transfected the following day with siRNA targeting PRC1 or with scrambled control siRNA. After 3 days, the cells were detached, seeded in 6-well plates and treated with 0 or 5 µM 3MBPP1. The colony formation ability was assessed 2 weeks later. The columns represent the means ± SEMs of 4 independent experiments performed in duplicate. *P < 0.05 vs control, Mann–Whitney test

Altered expression and mutation of PRC1 has been linked to human carcinogenesis^32–36^, while CDK16 is essential for the proliferation of some types of cancer cells^14^. A provocative hypothesis is that CDK16 exerts its proliferative effects via the regulation of PRC1. To examine the order of function, we used siRNA targeting PRC1^34^ to knock down PRC1 expression in 293T AS-CDK16 cells and/or applied 3MBPP1 to block AS-CDK16 activity (Fig. 6a).

Alone, both downregulation of PRC1 and inhibition of CDK16 significantly decreased cell viability, but the combination failed to induce a compound effect (Fig. 6b), consistent with a model in which CDK16 mediates its effects on cell proliferation via the regulation of PRC1. Similar results were obtained in the BrdU incorporation assay (Fig. 6c) and colony formation assay (Fig. 6d). Notably, the effect of PRC1 downregulation was more prominent on colony formation than in experiments with measurements at earlier time points (Fig. 6), suggesting that this effect may be time-dependent.

**Fig. 7:**
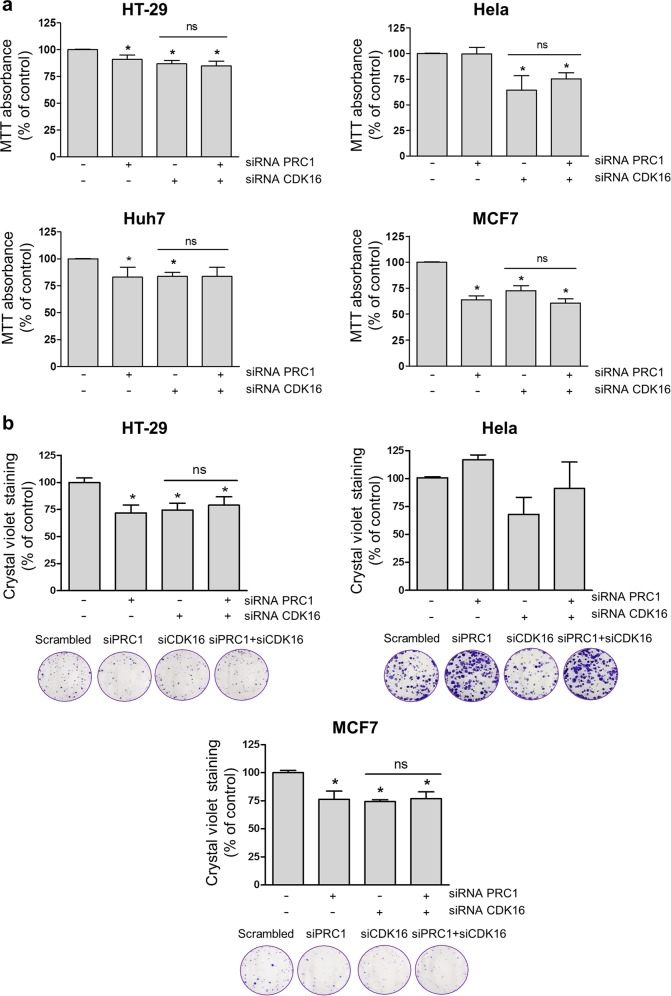
CDK16 promotes cancer cell proliferation via PRC1 phosphorylation. **a** Tumor cell lines were seeded and transfected the following day with siRNA targeting PRC1 and/or CDK16 or with scrambled control. Cell viability was assessed by an MTT assay, and the columns represent the means ± SEMs of at least 3 independent experiments performed in triplicate. *P < 0.05 vs control, Mann–Whitney test. **b** Tumor cell lines were seeded and transfected the following day with siRNA targeting PRC1 and/or CDK16 or with scrambled control. After 3 days, the cells were detached and seeded in 6-well plates. The colony formation ability was assessed 2 weeks later. The columns represent the means ± SEMs of at least 3 independent experiments performed in duplicate. *P < 0.05 vs control, Mann–Whitney test

Next, we investigated whether the effect of CDK16 on different types of cancer cell lines was also mediated via PRC1 phosphorylation. For this purpose, we used validated siRNA targeting CDK16^14^ and/or PRC1 to knock down the expression of these mediators. PRC1 downregulation decreased cell viability in all tested cancer cell lines except for HeLa cells, whereas CDK16 downregulation decreased cell viability in all cell lines (Fig. 7a). Consistent with previous results, downregulation of PRC1 and CDK16 failed to induce a compound effect on cell viability (Fig. 7a). To confirm these observations, we performed colony formation assays using the same cancer cell lines, with the exception of Huh7 cells, because we were unable to obtain a reliable number of colonies derived from this cell line under our experimental conditions. We observed that the effects of PRC1 and CDK16 downregulation on the colony formation ability were not additive (Fig. 7b), supporting the hypothesis that the proliferative action of CDK16 is mediated by PRC1 phosphorylation.

## Discussion

While CCNY has been shown to activate the kinase activity of CDK16^20,60^, physiological substrates of the CCNY/CDK16 complex remain unidentified. In the present study, using a dual proteomic approach, we discovered potential targets and partners of the CDK16/CCNY complex and validated PRC1 as a *bona fide* substrate. CDK16-dependent regulation of PRC1 localization and accumulation may mediate critical functions of both proteins in cell proliferation.

### Requirements for CDK16 activation

Our ability to detect the kinase activity of complexes formed from GST-CDK16 and GST-CCNY purified from bacteria confirms previous results^7^. The finding that CDK16/CCNY complexes may be active without CAK phosphorylation on the T-loop region suggests that binding of CCNY may be sufficient to activate CDK16, unlike for canonical CDK-cyclin complexes. In budding yeast, the PCL family cyclins bypass the need for CAK by encoding an aspartate residue that can activate the CDK5-related yeast CDK Pho85 by mimicking phosphorylation of the activation loop^61^. Electrostatic interactions between an aspartate residue in the PCL cyclins and nearby surface residues on Pho85, including salt bridges to arginine side chains, appear to mediate CAK independence. A similar geometry is present in the CDK5/p25 complex^61^. Aligning yeast Pcl1 to CCNY and Pho85 to CDK16 reveals conservation of the corresponding Asp and Arg residues, suggesting that this mechanism may be conserved and underlie the CAK independence of CDK16/CCNY.

A previous study by Sakamoto and colleagues^20^ showed that recombinant CDK16/CCNY complexes were active only in the presence of the 14-3-3 protein. Our results do not support this finding, but we note that we could only observe CDK16/CCNY activity after gel filtration to remove high molecular weight aggregates and enrich for heterodimers. Taken together, these findings indicate that aggregation may affect kinase activity, as previously suggested^10^, while binding of the 14-3-3 protein to CCNY may suppress the formation of multimers.

### CDK16/CCNY partners and substrates suggest a key role in cytoskeletal regulation

While affinity purification-mass spectrometry (AP-MS) has potential as a tool for the discovery of specific targets of a CDK, this approach faces several challenges, including the transient binding of kinases to their protein substrates^62^. Catalytically dead mutant kinases may display increased residence times^63,64^, and rapid processing^65^ may help preserve short-lived interactions. Alternatively, considering that the substrate specificity of a CDK is primarily conferred by its cyclin partner and that many CDKs can form active complexes with multiple cyclins, it may be preferable to perform pulldowns with cyclins instead. Whereas the conformation of CDKs changes dramatically upon cyclin binding, the substrate binding domains of cyclins appear to be comparatively rigid due to the compact cyclin fold. Thus, even if cyclins are not bound to their CDK partner(s), they may still capture specific interactors, including kinase substrates. Considering these observations, our initial strategy to discover substrates of CDK16/CCNY was to enrich by coimmunoprecipitation with an anti-CCNY antibody and identify putative CCNY-interacting proteins by LC–MS/MS.

In parallel, we developed an analog-sensitive mutant of CDK16 as an approach to performing a chemical genetic screen for substrates. Based on homology modeling, we identified phenylalanine 240 on CDK16 as a candidate gatekeeper residue and found that a complex formed from recombinant GST fusions to CDK16 F240A (AS-CDK16) and CCNY could transfer the thiophosphate from the bulky nucleotide N^6^-phenethyl ATPγS to CCNY. Thus, we screened for proteins in a cell lysate that could similarly be thiophosphorylated by the exogenous AS-CDK16/CCNY complexes but not by the endogenous kinases. This strategy has previously been applied to discover novel substrates of human CDK/cyclin complexes^48,66,67^. The 16 high-confidence thiophosphorylated peptides identified were sufficient to generate a consensus sequence for phosphorylation by CDK16 that can be summarized as S/T-P-positive-X-X-positive. In previous studies, Iwano et al.^9^ obtained a similar pattern from the phosphorylation of proteins in cells synchronized in mitosis, and Shehata et al.^20^ described a consistent consensus sequence based on the phosphorylation of artificial peptide substrates in an in vitro assay.

Notably, combining the lists generated by these two distinct approaches, many of the target candidates indicate an association with cytoskeleton-related pathways, with 7 out of 25 interactors being associated with the centrosome (Fig. 2a). These findings reveal a new role for the CCNY/CDK16 complex in the regulation of cytoskeletal metabolism, and considering the role of CDK16 in cell proliferation, it is tempting to speculate that CDK16 may be a pivotal element that couples progression through the cell cycle with cytoskeletal modifications.

Notably, PRC1 appears to be both a high-confidence CCNY interactor and a direct CDK16/CCNY substrate. The observation that PRC1 emerges as a high-confidence hit in two distinct cell lines, despite cancer type specificity and genetic variation, supports the relevance of our findings and suggests that the CDK16-PRC1 axis may be relevant in several cancer types. Therefore, we focused our attention on validating the role of PRC1 and examining the consequences of CDK16 phosphorylation.

### Potential roles for CDK16/CCNY in PRC1 regulation

PRC1 plays an important role in cytokinesis, which has been ascribed to its binding as a crosslinker to antiparallel spindle microtubules in the midzone. We observed PRC1 to be phosphorylated by CDK16/CCNY on T481 within a consensus NLS. Using the AS-CDK16 mutant and taking advantage of its increased sensitivity to the kinase inhibitor 3MBPP1, we found that PRC1 T481 phosphorylation appeared sufficient to limit PRC1 nuclear localization and reduce PRC1 accumulation. Notably, at a late time point (5 days of treatment), we were unable to detect PRC1 accumulation as a consequence of CDK inhibition (Fig. 6a), as we did in analyses performed after a 6 h treatment with 3MBPP1 (Fig. 5c). This result suggests that CDK inhibition leads to only transient accumulation of PRC1, likely reflecting the existence of other cellular mechanisms that tightly regulate the levels of this protein.

CDK16, CCNY and PRC1 are each known to play an oncogenic role^16,21,25,34,57,68^, which might be mediated by a common pathway. Consistent with this observation, the effects of PRC1 downregulation and CDK16 inhibition on cell viability were not additive. Interestingly, experiments using different cancer cell lines showed that the effect of PRC1 downregulation on cell proliferation varied significantly depending on the cell line, suggesting that the relevance of PRC1 to cell proliferation may be context-dependent.

What is the physiological relevance of PRC1 phosphorylation by CDK16? Although further investigation is needed, we propose a complex model of successive phosphorylation events that reconciles our results with those in previously published literature (see Li et al.^25^ for a revision). During the S and G2 phases, PRC1 is expressed and CDK16 activity increases^3,69^, which, coupled with CCNY accumulation^70^, leads to PRC1 phosphorylation on T481, restricting its nuclear localization. At the beginning of mitosis, the accumulation of the major mitotic CDK activity, CDK1/CCNB, may also contribute to PRC1 phosphorylation^27^. At that moment, when the nuclear membrane has been disassembled^71^, phosphorylation inhibits interaction with the Polo kinase^30,72^ and PRC1 dimerization^73^. While monomeric PRC1 still binds spindle microtubules, it can neither bundle them nor interact with Kif4A^28,74^, which is essential for midzone formation. With the increase in CDC14 phosphatase activity, PRC1 is dephosphorylated during the metaphase-to-anaphase transition, which enables its interaction with Kif4A^28^, and during anaphase, PRC1 is further regulated by Plk1-dependent phosphorylation and Plk1 binding, as previously described^30,72^.

The identification of PRC1 T481 as a target not only of CDK1/CCNB but also of CDK16/CCNY provides an example that counters the idea that each CDK/cyclin complex has its own specific substrate pattern and unique functions. Indeed, considering that the numbers of cyclins and CDKs increase with increasing organismal complexity, it seems reasonable that rather than reflecting an increased range of substrates and phosphosites, the diversity of cyclins and CDKs may facilitate regulation linked to a specific context, such as a developmental stage or particular tissue. While this model might appear as redundancy among CDKs and cyclins in some experiments, such a system is likely to provide a means to refine cell cycle regulation, with implications for normal development, cancer, aging and a wide range of degenerative diseases. These considerations may argue for a new approach to CDK inhibition that takes advantage of the distinct and overlapping functions of each CDK/cyclin complex to focus effects on specific physiological or pathological targets.

## Supplementary information

Supplementary Information
