# Supplementary material for: Phosphoregulation of the oncogenic protein regulator of cytokinesis 1 (PRC1) by the atypical CDK16/CCNY complex
Source: Exp Mol Med. 2019 Apr 16;51(4):44. doi: 10.1038/s12276-019-0242-2 (PMC6467995; doi:10.1038/s12276-019-0242-2)
Supplement: Supplementary file 1 — Supplementary Information [file 12276_2019_242_MOESM1_ESM.pdf]

# Supplementary Information

# Phosphoregulation of the Oncogenic Protein Regulator of Cytokinesis 1 (PRC1) by the Atypical CDK16/CCNY Complex

Sara Hernández-Ortega[&], Abril Sánchez-Botet[&], Eva Quandt, Núria Masip, Laura Gasa, Gaetano Verde, Javier Jiménez, Rebecca S. Levin, Florentine U. Rutaganira, Alma L. Burlingame, Don Wolfgeher, Mariana P.C. Ribeiro[*], Stephen J. Kron, Kevan M. Shokat and Josep Clotet[*].

[&]Contributed equally
*Corresponding authors

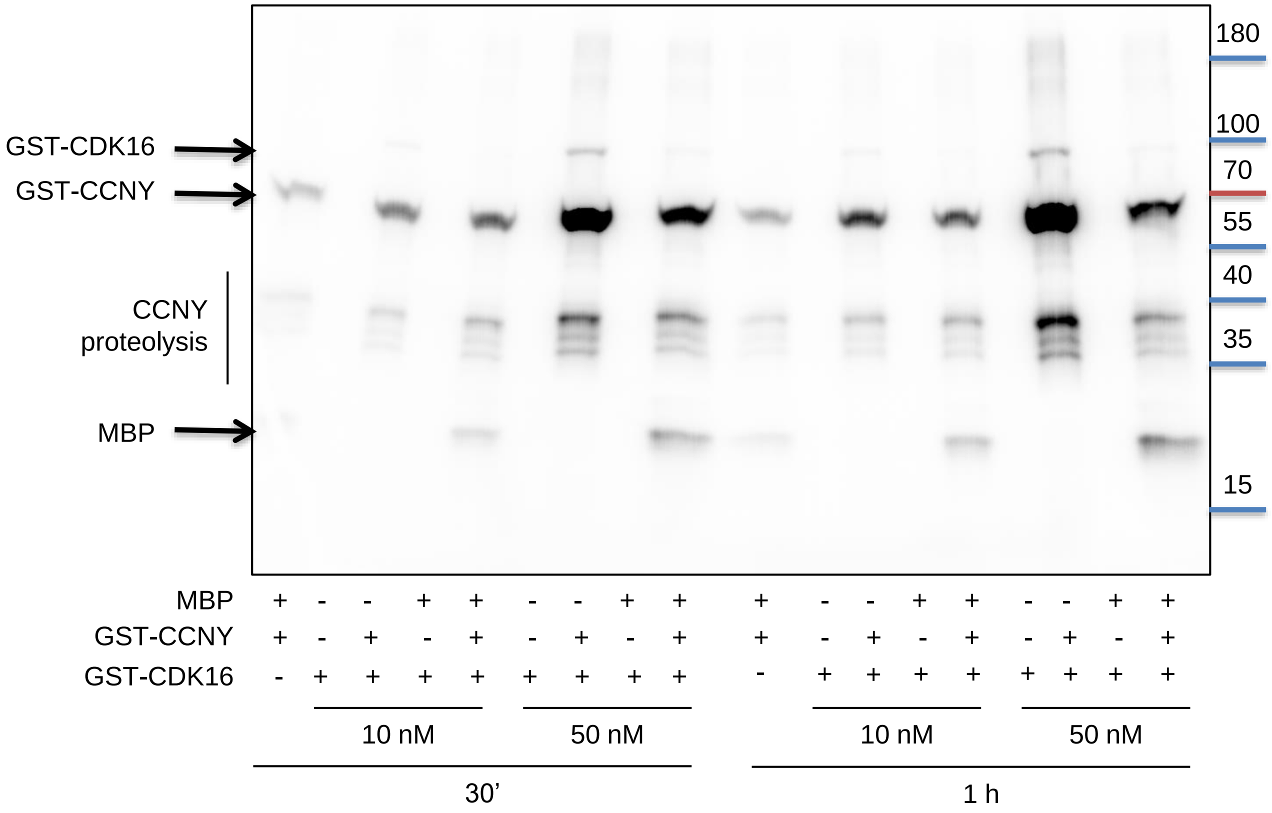


**Supplementary Figure S1. CDK16/CCNY phosphorylates MBP in vitro.** GST-CDK16 and GST-CCNY were purified from *E. Coli* and MBP was purchased from Millipore. Proteins were incubated with [γ-$^{32}$P]-ATP in the presence of CDK16 (at 10 or 50 nM) for the indicated time interval.

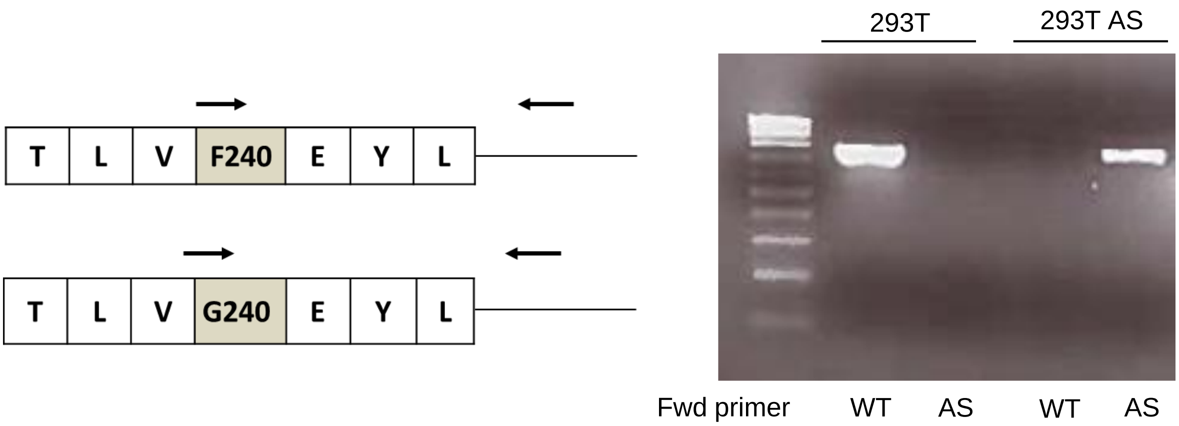


**Supplementary Figure S2. The insertion of the F240G mutation in 293T cells using CRISPR/Cas9 system was confirmed by PCR.** The strategy designed to confirm the introduction of the desired point mutation is shown. Each colony was genotyped by PCR using a forward primer specific for either the wild-type (WT) or the mutated locus (AS). Diagrams above each set of reactions indicate the relative positions of the primer pairs.

**a**

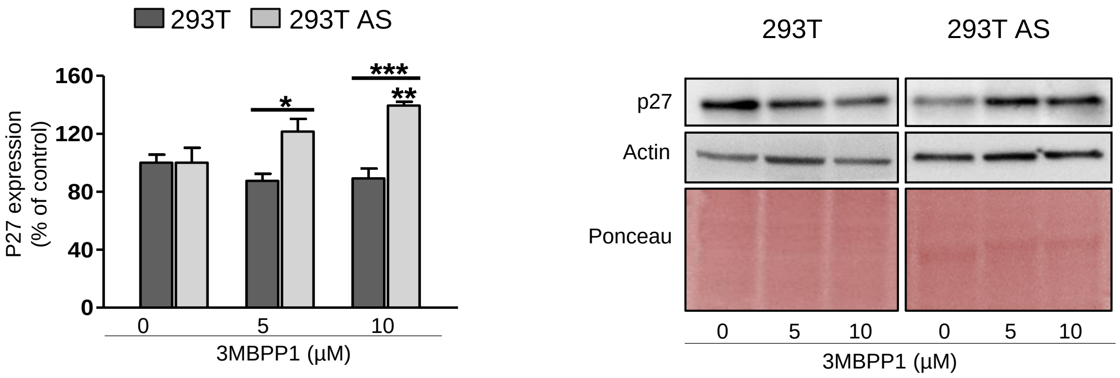


**b**

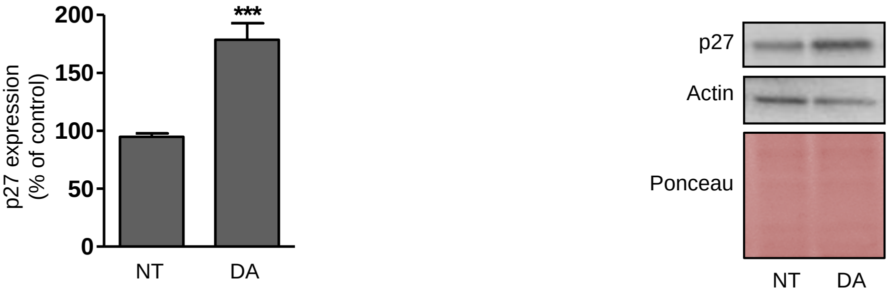


**Supplementary Figure S3. CDK16 inhibition promotes p27 accumulation.** (A) The expression of p27 was monitored in 293T and 293T AS-CDK16 cells following treatment with 3MBPP1 for 6 h. Columns represent the mean ± SEM of 12 independent experiments. *P < 0,05, **P < 0.01, ***P < 0.001 vs non-treated cells, Mann-Whitney test. (B) HT-29 colon cells were treated with 2 nM of dabrafenib (DA) for 24 h.The accumulation of p27 was monitored by western blot. Columns represent the mean ± SEM of 8 independent experiments. ***P < 0.001 vs non-treated (NT) cells, Mann-Whitney test.

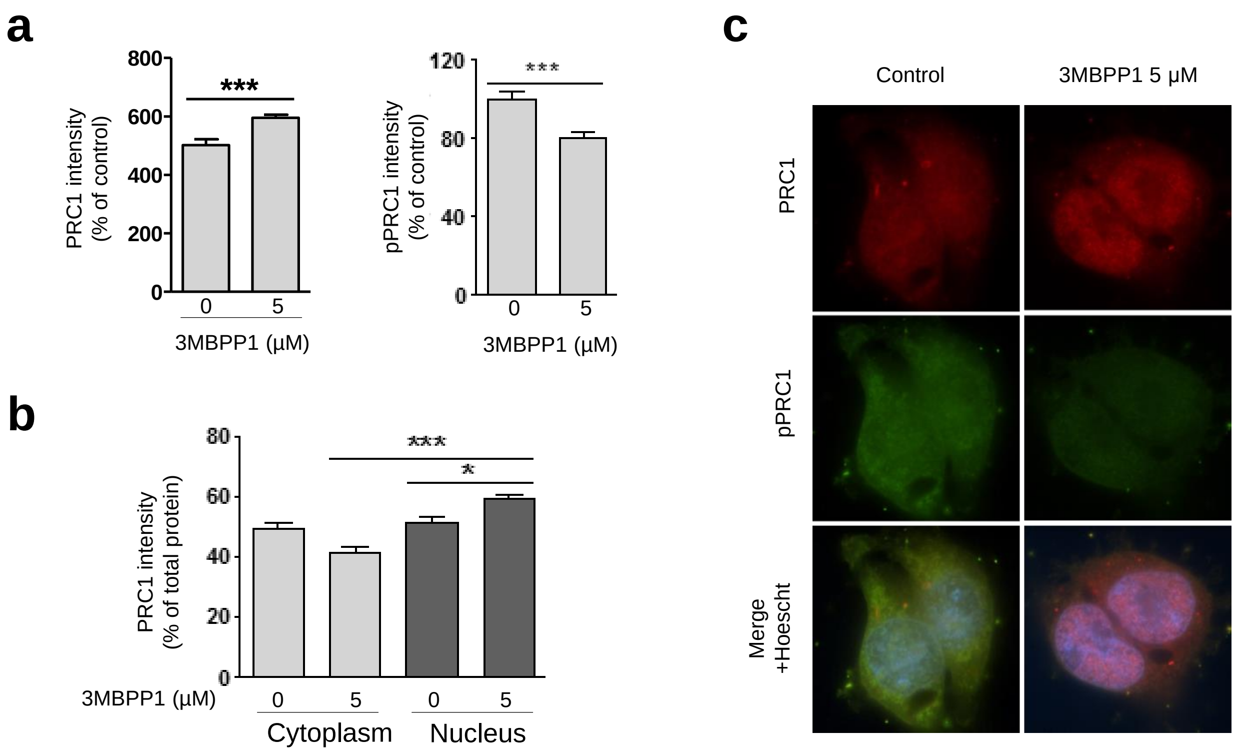


**Supplementary Figure S4. CDK16 inhibition decreases PRC1 phosphorylation and leads to PRC1 accumulation and nuclear trans-localization.** (a) The expression of PRC1 and phospho-PRC1 was monitored in 293T AS-CDK16 cells following treatment with 3MBPP1. ***P < 0.001 vs non-treated cells, Student's t-test. (b) PRC1 delocalization to the nucleus following treatment with 3MBPP1 was monitored by immunofluorescence. *P < 0,05, ***P < 0.001 vs non-treated cells, Student's t-test. (c) Representative image of immunofluorescence studies demonstrating the decrease in PRC1 phosphorylation and nuclear accumulation.

**a**

| Gene Name | Protein Name | ID | Log2(CCNY/C-) | P-value |
|---|---|---|---|---|
| CCNY | Cyclin-Y | Q8ND76 | 12,00 | 1,23E-05 |
| WDR37 | WD repeat-containing protein 37 | Q9Y2I8 | 11,70 | 4,46E-07 |
| ZCCHC8 | Zinc finger CCHC domain-containing protein 8 | Q6NZY4 | 11,30 | 4,11E-06 |
| CCNYL1 | Cyclin-Y-like protein 1 | Q8N7R7 | 11,00 | 1,13E-06 |
| PCNT | Pericentrin | O95613 | 9,69 | 2,80E-06 |
| SKIV2L2 | Superkiller viralicidic activity 2-like 2 | P42285 | 9,04 | 9,15E-05 |
| **PRC1** | **Protein regulator of cytokinesis 1** | **O43663-4** | **8,54** | **3,23E-04** |
| PACS1 | Phosphofurin acidic cluster sorting protein 1 | Q6VY07 | 8,40 | 1,15E-03 |
| TJP1 | Tight junction protein ZO-1 | Q07157-2 | 7,34 | 2,15E-04 |
| CDKN2AIP | CDKN2A-interacting protein | Q9NXV6 | 7,24 | 1,57E-04 |
| PACSIN2 | Protein kinase C and casein kinase substrate in neurons protein 2 | Q9UNF0-2 | 7,08 | 3,55E-03 |
| PACS2 | Phosphofurin acidic cluster sorting protein 2 | Q86VP3-4 | 6,52 | 2,61E-05 |
| ATG7 | Ubiquitin-like modifier-activating enzyme ATG7 | O95352 | 6,50 | 3,87E-03 |
| CDK5RAP2 | CDK5 regulatory subunit-associated protein 2 | Q96SN8-3 | 6,45 | 9,39E-05 |
| TERF2 | Telomeric repeat-binding factor 2 | Q15554 | 5,53 | 3,17E-03 |
| PCM1 | Pericentriolar material 1 protein | Q15154-2 | 5,47 | 2,80E-03 |
| CDC42BPG | Serine/threonine-protein kinase MRCK gamma | Q6DT37 | 5,46 | 2,89E-02 |
| XRN2 | 5-3 exoribonuclease 2 | Q9H0D6-2 | 5,36 | 2,02E-05 |
| SPAST | Spastin | Q9UBP0-3 | 5,34 | 4,66E-03 |
| MIB1 | E3 ubiquitin-protein ligase MIB1 | Q86YT6 | 5,16 | 7,80E-04 |
| UFD1L | Ubiquitin fusion degradation protein 1 homolog | Q92890-3 | 4,42 | 1,11E-02 |
| CEP131 | Centrosomal protein of 131 kDa | Q9UPN4-3 | 4,38 | 1,72E-02 |
| GOLGB1 | Golgin subfamily B member 1 | Q14789-4 | 4,08 | 2,25E-02 |
| PPP1R12A | Protein phosphatase 1 regulatory subunit 12A | O14974-5 | 4,03 | 3,91E-02 |
| YWHAH | 14-3-3 protein eta | Q04917 | 3,23 | 2,14E-02 |
| PRKCSH | Glucosidase 2 subunit beta | P14314-2 | -2,59 | 3,02E-02 |
| SH3GLB2 | Endophilin-B2 | Q9NR46 | -3,05 | 5,26E-03 |
| CRNN | Cornulin | Q9UBG3 | -3,45 | 9,20E-03 |
| DIP2B | Disco-interacting protein 2 homolog B | Q9P265 | -3,55 | 2,24E-02 |
| FTH1 | Ferritin heavy chain;Ferritin heavy chain, N-terminally processed | P02794 | -3,67 | 4,26E-02 |
| ATP1A1 | Sodium/potassium-transporting ATPase subunit alpha-1 | P05023-2 | -4,55 | 2,96E-03 |
| NCOA5 | Nuclear receptor coactivator 5 | Q9HCD5 | -4,96 | 4,79E-02 |
| SHMT1 | Serine hydroxymethyltransferase, cytosolic | P34896-2 | -5,86 | 3,51E-03 |
| FBP1 | Fructose-1,6-bisphosphatase 1 | P09467 | -6,71 | 2,73E-05 |

**b**

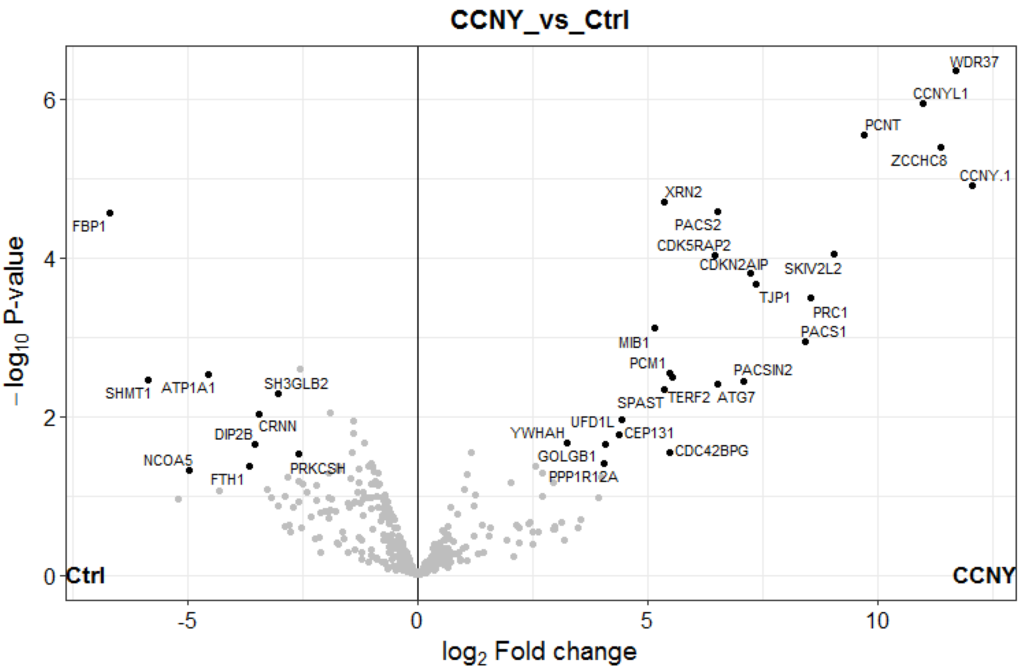


**Supplementary Table S1. List of CCNY interactors.** (a) Data was filtered at a p-value cutoff of 0.05 and fold change cutoff of $Log_2(3.23)$ and seen in all three biological replicates. (b) Volcano plot representing the hits obtained after screening A (CCNY interactome).

| Gene Name | Protein Name | ID | Unique peptides |
|---|---|---|---|
| CDK16 | Cyclin-dependent kinase 16 | Q00536 | 25 |
| CCNY | Cyclin-Y | Q8ND76 | 13 |
| EEF2 | Elongation factor 2 | P13639 | 6 |
| CEP170 | Centrosomal protein of 170 kDa | Q5SW79 | 5 |
| NUCKS1 | Nuclear ubiquitous casein and cyclin-dependent kinase substrate 1 | Q9H1E3 | 4 |
| EPB41L2 | Band 4.1-like protein 2 | O43491 | 3 |
| PHLDB1 | Pleckstrin homology-like domain family B member 1 | Q86UU1 | 3 |
| TOR1AIP1 | Torsin-1A-interacting protein 1 | Q5JTV8 | 3 |
| BOD1L1 | Biorientation of chromosomes in cell division protein 1-like 1 | Q8NFC6 | 3 |
| **PRC1** | **Protein regulator of cytokinesis 1** | **O43663** | **3** |
| LARP1 | La-related protein 1 | Q6PKG0 | 3 |
| ERC1 | ELKS/Rab6-interacting/CAST family member 1 | Q8IUD2 | 3 |
| SRRM2 | Serine/arginine repetitive matrix protein 2 | Q9UQ35 | 2 |
| TPR | Nucleoprotein TPR | P12270 | 2 |
| MAP4 | Microtubule-associated protein 4 | P27816 | 2 |
| DPM1 | Dolichol-phosphate mannosyltransferase | O60762 | 2 |
| KIF21A | Kinesin-like protein KIF21A | Q7Z4S6 | 2 |
| XRCC1 | DNA repair protein XRCC1 | P18887 | 2 |
| MYCBP2 | Probable E3 ubiquitin-protein ligase MYCBP2 | O75592 | 2 |
| PHLDB2 | Pleckstrin homology-like domain family B member 2 | Q86SQ0 | 2 |
| SPATA31E1 | Spermatogenesis-associated protein 31E1 | Q6ZUB1 | 1 |
| SSH3 | Protein phosphatase Slingshot homolog 3 | Q8TE77 | 1 |
| AGAP1 | Arf-GAP with GTPase, ANK repeat and PH domain-containing protein 1 | Q9UPQ3 | 1 |
| PM20D1 | Probable carboxypeptidase PM20D1 | Q6GTS8 | 1 |
| PRRC2A | Protein PRRC2A | P48634 | 1 |
| EML4 | Echinoderm microtubule-associated protein-like 4 | Q9HC35 | 1 |
| DENND4C | DENN domain-containing protein 4C | Q5VZ89 | 1 |
| C4orf21 | Uncharacterized protein C4orf21 | Q86YA3 | 1 |
| ALDH1L2 | Mitochondrial 10-formyltetrahydrofolate dehydrogenase | Q3SY69 | 1 |
| FGD5 | FYVE, RhoGEF and PH domain-containing protein 5 | Q6ZNL6 | 1 |
| PAN3 | PAB-dependent poly(A)-specific ribonuclease subunit 3 | Q58A45 | 1 |
| SYNPO | Synaptopodin | Q8N3V7 | 1 |
| NUP98 | Nuclear pore complex protein Nup98-Nup96 | P52948 | 1 |
| CTTN | Src substrate cortactin | Q14247 | 1 |
| CCDC6 | Coiled-coil domain-containing protein 6 | Q16204 | 1 |
| HTATSF1 | HIV Tat-specific factor 1 | O43719 | 1 |
| TSC22D4 | TSC22 domain family protein 4 | Q9Y3Q8 | 1 |
| ARHGEF12 | Rho guanine nucleotide exchange factor 12 | Q9NZN5 | 1 |
| CDR2 | Cerebellar degeneration-related protein 2 | Q01850 | 1 |
| PDE3A | cGMP-inhibited 3',5'-cyclic phosphodiesterase A | Q14432 | 1 |
| PNN | Pinin | Q9H307 | 1 |
| ABL2 | Abelson tyrosine-protein kinase 2 | P42684 | 1 |
| RTN3 | Reticulon-3 | O95197 | 1 |
| ABL1 | Tyrosine-protein kinase ABL1 | P00519 | 1 |
| NUP35 | Nucleoporin NUP53 | Q8NFH5 | 1 |
| BCAR3 | Breast cancer anti-estrogen resistance protein 3 | O75815 | 1 |
| TPX2 | Targeting protein for Xklp2 | Q9ULW0 | 1 |
| DNMT1 | DNA (cytosine-5)-methyltransferase 1 | P26358 | 1 |
| FAM195A | Protein FAM195A | Q9BUT9 | 1 |
| UNG | Uracil-DNA glycosylase | P13051 | 1 |

**Supplementary Table S2**. **List of Cdk16/CCNY substrates.** To be selected, peptides had to be detected in the AS-CDK16 proteomic, but neither in the WT-CDK16 nor in the negative control. Shaded rows indicate high confidence CDK16 substrates (only those that present a number of unique peptides ≥2 and that were not found in other proteomic screenings using AS models).
